# Massive dominance of *Epsilonproteobacteria* in formation waters from a Canadian oil sands reservoir containing severely biodegraded oil

**DOI:** 10.1111/j.1462-2920.2011.02521.x

**Published:** 2012-02

**Authors:** Casey R J Hubert, Thomas B P Oldenburg, Milovan Fustic, Neil D Gray, Stephen R Larter, Kevin Penn, Arlene K Rowan, Rekha Seshadri, Angela Sherry, Richard Swainsbury, Gerrit Voordouw, Johanna K Voordouw, Ian M Head

**Affiliations:** 1 School of Civil Engineering and Geosciences, Newcastle UniversityNewcastle upon Tyne NE1 7RU, UK; 2 Petroleum Reservoir Group, Department of Geoscience, University of CalgaryCalgary, Canada, T2N 1N4; 3 J. Craig Venter InstituteSan Diego, CA 92121, USA; 4 Department of Biological Sciences, University of CalgaryCalgary, Canada, T2N 1N4

## Abstract

The subsurface microbiology of an Athabasca oil sands reservoir in western Canada containing severely biodegraded oil was investigated by combining 16S rRNA gene- and polar lipid-based analyses of reservoir formation water with geochemical analyses of the crude oil and formation water. Biomass was filtered from formation water, DNA was extracted using two different methods, and 16S rRNA gene fragments were amplified with several different primer pairs prior to cloning and sequencing or community fingerprinting by denaturing gradient gel electrophoresis (DGGE). Similar results were obtained irrespective of the DNA extraction method or primers used. Archaeal libraries were dominated by *Methanomicrobiales* (410 of 414 total sequences formed a dominant phylotype affiliated with a *Methanoregula* sp.), consistent with the proposed dominant role of CO_2_-reducing methanogens in crude oil biodegradation. In two bacterial 16S rRNA clone libraries generated with different primer pairs, > 99% and 100% of the sequences were affiliated with *Epsilonproteobacteria* (*n* = 382 and 72 total clones respectively). This massive dominance of *Epsilonproteobacteria* sequences was again obtained in a third library (99% of sequences; *n* = 96 clones) using a third universal bacterial primer pair (inosine-341f and 1492r). Sequencing of bands from DGGE profiles and intact polar lipid analyses were in accordance with the bacterial clone library results. Epsilonproteobacterial OTUs were affiliated with *Sulfuricurvum*, *Arcobacter* and *Sulfurospirillum* spp. detected in other oil field habitats. The dominant organism revealed by the bacterial libraries (87% of all sequences) is a close relative of *Sulfuricurvum kujiense –* an organism capable of oxidizing reduced sulfur compounds in crude oil. Geochemical analysis of organic extracts from bitumen at different reservoir depths down to the oil water transition zone of these oil sands indicated active biodegradation of dibenzothiophenes, and stable sulfur isotope ratios for elemental sulfur and sulfate in formation waters were indicative of anaerobic oxidation of sulfur compounds. Microbial desulfurization of crude oil may be an important metabolism for *Epsilonproteobacteria* indigenous to oil reservoirs with elevated sulfur content and may explain their prevalence in formation waters from highly biodegraded petroleum systems.

## Introduction

**Fig 1:**
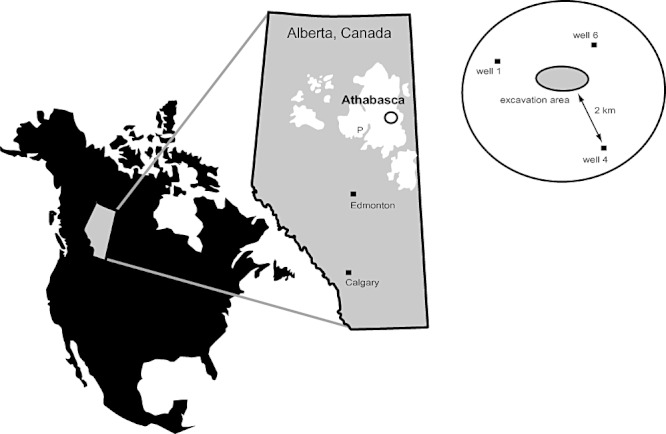
Geographic location of the Athabasca oil sands. Three formation water samples were taken from around the Muskeg River open pit mine site where unconventional heavy oil recovery operations take place. The approximate location of the Pelican Lake oil reservoir where conventional recovery is practised (Grabowski *et al*., 2005a,b) is also indicated (P).

The global inventory of petroleum reserves is dominated by heavy oil reservoirs that represent a legacy of anaerobic microbial communities that have degraded hydrocarbons *in situ* over geological timescales (Head *et al*., 2003; 2010; Larter *et al*., 2006). These biologically altered fossil fuels represent an enormous energy resource, yet their production is complicated by detrimental factors such as increased oil viscosity, acidity and sulfur content. Extreme cases of biodegradation in ‘unconventional’ super-heavy oil reservoirs have led to the development of novel extraction techniques involving mining or steam injection for producing this petroleum. A prominent example is the Athabasca oil sands (Fig. 1), where increased productionin recent years underscores Canada having the second largest proven domestic crude oil reserves worldwide (*c*. 170 billion barrels; ERCB, 2009). The many challenges associated with producing heavy oil or bitumen (highly viscous oil) translate into added costs and increasing environmental impacts, and highlight the ongoing need for innovation in the oil sands sector. Biotechnologies based on understanding and manipulating subsurface reservoir microbial communities have been proposed as a promising route towards more sustainable production of energy from fossil fuels including oil sands (Grigoryan and Voordouw, 2008; Jones *et al*., 2008; Youssef *et al*., 2009). These strategies are underpinned by a view of petroleum reservoirs as important habitats within the ‘deep biosphere’. Investigating reservoir habitats is often enabled by sampling produced water, which has been conducted for several oil fields including some in western Canada (Voordouw *et al*., 1996; Grabowski *et al*., 2005a). Similar studies from unconventional oil sands reservoirs have not been reported to date.

The occurrence of fermentative bacteria and methanogenic archaea in several oil fields (Jeanthon *et al*., 2005; Ollivier and Cayol, 2005) befits current models of anaerobic hydrocarbon degradation in subsurface petroleum reservoirs. Hydrocarbon geochemistry and gas isotope data have highlighted crude oil hydrocarbon degradation dominated by CO_2_ reduction to methane as an important mechanism (Jones *et al*., 2008; Gray *et al*., 2009; 2010). Experimental microcosms inoculated with surface sediments show that anaerobic degradation of hydrocarbons and crude oil can be catalysed by consortia dominated by hydrogenotrophic (CO_2_-reducing) methanogens (e.g. order *Methanomicrobiales*) and fermentative syntrophic *Deltaproteobacteria* (e.g. family *Syntrophaceae*) (Zengler *et al*., 1999; Jones *et al*., 2008). This hypothesis is consistent with the majority of the methanogenic archaea recovered so far from oil fields being hydrogenotrophic CO_2_ reducers (Jeanthon *et al*., 2005; Nazina *et al*., 2006; Gray *et al*., 2009). Interestingly, bacterial community analyses of reservoir samples have not revealed a clear prominence of *Syntrophaceae* to mirror the widespread occurrence of *Methanomicrobiales*. The absence, or low abundance of these putative syntrophs in oil reservoir communities may indicate that other bacterial groups might fill this niche in subsurface petroleum ecosystems. An alternative explanation could be the relatively few investigations of indigenous microbial communities from actively biodegrading oil reservoirs where syntrophs are expected to be important *in situ*. Grabowski and colleagues (2005a,b) examined formation waters (i.e. co-produced with crude oil during primary recovery operations prior to secondary water flooding) from the Pelican Lake oil field – a 400 m deep conventional heavy oil reservoir in west Athabasca (Fig. 1). Fatty acid-degrading *Syntrophus* spp. could be enriched from this formation water after several transfers (Grabowski *et al*., 2005b), but were not detected in a bacterial 16S rRNA gene clone library constructed from DNA extracted from formation water (Grabowski *et al*., 2005a). This clone library consisted of 151 sequences from a single taxon affiliated with the genus *Arcobacter* within the *Epsilonproteobacteria* (Grabowski *et al*., 2005a).

Even though *Epsilonproteobacteria* have been detected in several oil fields using a variety of methods (e.g. Voordouw *et al*., 1996; Telang *et al*., 1997; Gevertz *et al*., 2000; Grabowski *et al*., 2005a; Hubert and Voordouw, 2007; Sette *et al*., 2007; Gittel *et al*., 2009; Pham *et al*., 2009), the unexpected predominance of sequences from *Epsilonproteobacteria* in the Pelican Lake clone library has led to the suggestion that this may represent an artefact arising from selective amplification of epsilonproteobacterial 16S rRNA gene sequences by certain PCR primers (Grabowski *et al*., 2005a; Pham *et al*., 2009; Head *et al*., 2010). Such primer selectivity was shown to explain similar results obtained by Watanabe and colleagues (2000) who reported strong dominance of *Epsilonproteobacteria* in a similar environment (91% of clones derived from groundwater in an underground crude oil storage cavity), but later reported that using different PCR primers resulted in recovery of a greater diversity of sequences and a smaller proportion affiliated with *Epsilonproteobacteria* (no more than 16% of clones; Watanabe *et al*., 2002).

Here we present a microbiological characterization of formation waters from an unconventional shallow oil sands reservoir (0–80 m) from the northern Athabasca region in western Canada where the oil is severely degraded and immobile. Geochemical, microbiological, and lipid- and DNA-based molecular analyses of crude oil and formation water were performed, including PCR-based screening of 16S rRNA gene sequences from resident *Archaea* and *Bacteria* by employing different DNA extraction and PCR protocols to circumvent selectivity imposed by using only a single approach. The results reveal a strong dominance of *Epsilonproteobacteria* in the formation water samples from this oil sands reservoir. Coupling this observation with geochemical data showing that dibenzothiophenes are extensively biodegraded throughout the oil column and particularly at the oil water transition zone, we suggest that the predominance of *Epsilonproteobacteria* may be explained by the ability of these bacteria to use organic sulfur compounds in crude oil as an electron donor and energy source (Kodama and Watanabe, 2003).

## Results

### Site description and formation water geochemistry

Samples were obtained from the Muskeg River mine, located approximately 75 km north of the city of Fort McMurray, Alberta (Fig. 1). In this oil sands reservoir, formation water occurs as a basal aquifer up to 20 m in thickness underlying a 50–80 m thick layer of oil-saturated sands. Formation waters are therefore not in contact with surface waters. Muskeg River oil sands are produced by clearing several metres of topsoil (overburden) to allow large-scale truck and shovel operation to excavate and transport oil sands to nearby facilities that separate the bitumen from the sand. This unconventional surface mining approach requires advanced dewatering of the oil sands to reduce formation pressure and prevent fractures and flooding as the open pit excavation proceeds deeper into the reservoir. Therefore formation waters are discharged at dewatering wells located 500–2500 m ahead of the advancing production area. Discharged formation waters should thus derive from biologically active oil water transition zones and associated aquifers in the subsurface (Head *et al*., 2003; Larter *et al*., 2003) and represent pristine reservoir samples unaffected by any prior water or chemical injections that sometimes characterize produced water samples from deeper conventional oil fields (Magot, 2005). Waters discharged from six wells around the mine excavation area had a combined flow rate of 250 m^3^ h^−1^, and three of these wells were sampled for geochemical and microbiological analyses (Fig. 1). The formation water geochemistry is summarized in Table 1, and indicates that the three samples are broadly similar. Similar microbial community compositions in the three water samples were confirmed by denaturing gradient gel electrophoresis (DGGE) (not shown) and intact polar lipid (IPL) analyses (Oldenburg *et al*., 2009). Further community analyses focused on formation water from wellhead #1.

**Table 1:** Geochemistry of oil sands formation water samples.

Parameter	Wellhead #1	Wellhead #4	Wellhead #6
Basal aquifer thickness	18.6 m	17.6 m	23.0 m
Cumulative discharge at the time of sampling	227 000 m^3^	373 000 m^3^	387 000 m^3^
Discharge flow rate (flow continuity)	10–20 m^3^ h^−1^ (continuous)	20–30 m^3^ h^−1^ (semi-continuous)	60–70 m^3^ h^−1^ (semi-continuous)
Electrical conductivity mS cm^−1^	2.36	5.22	4.20
pH	7.20	7.38	7.43
Alkalinity (mg l^−1^ HCO_3_^-^)	959.3	1512.0	1387.0
Intact polar lipids detected	Phosphatidylethanolamine; phosphatidylglycerol	Phosphatidylethanolamine; phosphatidylglycerol	Phosphatidylethanolamine; phosphatidylglycerol
δ^34^S elemental S (‰)	ND	26.7	26.7
δ^34^S SO_4_^2−^ (‰)	22.8	23.6	23.4
SO_4_^2−^ (mg l^−1^)	15.37	32.50	23.03
NO_3_^-^	ND	ND	ND
PO_4_^2−^	ND	ND	ND
Mn^2+^	0.27	0.04	0.02
Fe^2+^	0.50	0.15	0.10
Na^+^	454.00	1276.00	1033.10
Cl^-^	351.22	1226.11	877.24
Br^-^	5.71	9.68	8.60
F^-^	3.23	3.60	3.72
Si^+^	9.24	4.33	5.48
Ba^2+^	0.81	0.16	0.22
Sr^2+^	2.04	1.84	1.63
Li^+^	0.22	0.25	0.22
Ca^2+^	77.07	51.11	52.78
Mg^2+^	43.42	31.84	30.70
K^+^	18.10	22.10	19.40

### Formation water microbial community composition

Two archaeal clone libraries (one prepared in Newcastle and the other in Calgary and Rockville) and three bacterial clone libraries (two prepared in Newcastle and one in Calgary and Rockville) were constructed from DNA that was extracted from the biomass that had been concentrated by filtering 12 l of formation water from wellhead #1. The diversity in all libraries was low with just seven archaeal and five bacterial phylotypes being recovered from the five libraries (Table 2; operational taxonomic units (OTUs) were defined at 97% sequence identity). Clone library results indicate that regardless of DNA extraction procedure or PCR primers used, similar community compositions were obtained for *Archaea* and *Bacteria* in the oil sands formation water. The Newcastle archaeal library (91 clones) consisted of just a single phylotype affiliated with *Methanoregula* (Table 2). This phylotype was also dominant (89% of clones) in the larger J. Craig Venter Institute (JCVI) library (*n* = 323 clones analysed), which contained additional phylotypes related to *Methanospirillum* (9% of clones), unclassified *Methanomicrobiales* (0.6%), *Methanosarcina* (0.3%), *Desulfurococcales* (0.3%) and *Thermoplasmatales* (0.3% for each of two distinct OTUs) (Table 2).

**Table 2:** Archaeal and bacterial 16S rRNA gene clone library results.

Library; *target group* (16S primers) (DNA extraction)	No. of clones sequenced in total	Lineages of phylotypes detected	% abundance in library (No. of clones)	Representative type sequence	Accession No.
**A1***Archaea* (arch8f/arch1492r) (Calgary)	323	*Methanoregula*	88.9% (287)	TS1A275	JF789587
		*Methanospirillum*	9.3% (30)	TS1A121	JF789588
		*Methanomicrobiales*	0.6% (2)	TS1A142	JF789589
		*Methanosarcina*	0.3% (1)	TS1A083	JF789590
		*Desulfurococcales*	0.3% (1)	TS1A038	JF789591
		*Thermoplasmatales* 1	0.3% (1)	TS1A251	JF789592
		*Thermoplasmatales* 2	0.3% (1)	TS1A042	JF789593
**A2***Archaea*(arch46f/arch1017r) (Newcastle)	91	*Methanoregula*	100% (91)	(TS1A275)^a^	(JF789587)
**B1***Bacteria* (9f/1545r)(Calgary)	382	*Sulfuricurvum*	95.6% (369)	TS1B301	JF789594
		*Arcobacter*	2.9% (11)	TS1B220	JF789595
		*Sulfurospirillum* 1	0.3% (1)	TS1B252	JF789596
		*Pelobacter*	0.3% (1)	TS1B322	JF789597
**B2***Bacteria* (8f/1542r) (Newcastle)	72	*Sulfuricurvum*	63.8% (46)	(TS1B301)	(JF789594)
		*Arcobacter*	31.9% (23)	(TS1B220)	(JF789595)
		*Sulfurospirillum* 2	4.2% (3)	NCL08_D6E05	JF789598
**B3***Bacteria*(ino-341f/1492r) (Newcastle)	96	*Sulfuricurvum*	63.5% (61)	(TS1B301)	(JF789594)
		*Arcobacter*	29.2% (28)	(TS1B220)	(JF789595)
		*Sulfurospirillum* 2	6.3% (6)	(NCL08_D6E05)	(JF789598)
		*Pelobacter*	1.0% (1)	(TS1B322)	(JF789597)

a Designations in parentheses indicate the type sequence is from one of the other libraries.

Common to all three bacterial libraries were two phylotypes related to *Sulfuricurvum* and *Arcobacter*, with *Sulfuricurvum* always being the most abundant group (Table 2). *Arcobacter* comprised a much higher proportion of clones in Newcastle libraries, which were constructed from DNA that was extracted using a bead beating method (30% *Arcobacter* clones in Newcastle libraries as opposed to 3% in the JCVI library; Table 2). Both of the Newcastle libraries revealed a distinct *Sulfurospirillum* phylotype (4% and 6% of clones) that was not detected in the larger JCVI library, and the JCVI library contained a distinct *Sulfurospirillum* phylotype (a singleton) that was not detected in the Newcastle libraries. The only non-epsilonproteobacterial clones were singletons from the two larger libraries that comprised a fourth phylotype related to *Pelobacter* in the *Deltaproteobacteria*.

Only two bacterial clone libraries were constructed initially, using primers 9f/1545r and 8f/1542r (Table S1), resulting in 99.7% and 100% of the cloned 16S rRNA gene sequences being affiliated with *Epsilonproteobacteria* (Table 2). This was unexpected, and similar to the 91% dominance of *Epsilonproteobacteria* in an underground storage cavity reported by Watanabe and colleagues (2000) using a similar primer pair (8f/1546r). Watanabe and colleagues (2002) subsequently constructed an additional library using an inosine-substituted 341f primer together with 1492r, which resulted in only 16% of clones affiliated with *Epsilonproteobacteria* (also related to *Sulfuricurvum*, *Arcobacter* and *Sulfurospirillum*). However, when the primers designed by Watanabe and colleagues (2002) were used to construct a third bacterial clone library from the oil sands formation water, again 99% of sequences were affiliated with *Epsilonproteobacteria* (Table 2).

**Fig 2:**
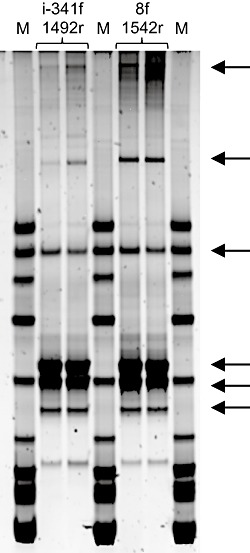
Denaturing gradient gel electrophoresis of bacterial 16S rRNA gene fragments following two-step nested PCR. Amplification with different universal bacterial primers (indicated above gel lanes; see also Table S1) was followed by a second round of amplification using DGGE primers to create amplicons 233 base pairs in length (including a 40-base-pair GC clamp) for DGGE, as described in the text. Arrows indicate bands from lanes 2–3 and 5–6 that were excised and sequenced. All sequences were closely related to the *Sulfuricurvum* OTU detected in the bacterial 16S rRNA gene clone libraries.

The bacterial community composition determined by the three clone libraries was confirmed using a nested DGGE strategy whereby different universal bacterial primer pairs 8f/1542r or inosine-341f/1492r were used to amplify 16S rRNA genes from the formation water DNA sample, with resulting PCR products used as a template for DGGE-PCR using 341f-GC/534r (Table S1). Figure 2 shows that both methods resulted in the same banding pattern. All bands that were excised and sequenced corresponded to *Epsilonproteobacteria*, having 97–100% sequence identity to the dominant *Sulfuricurvum* phylotype from the clone libraries (data not shown).

### *Archaea* detected in oil sands formation waters

**Fig 3:**
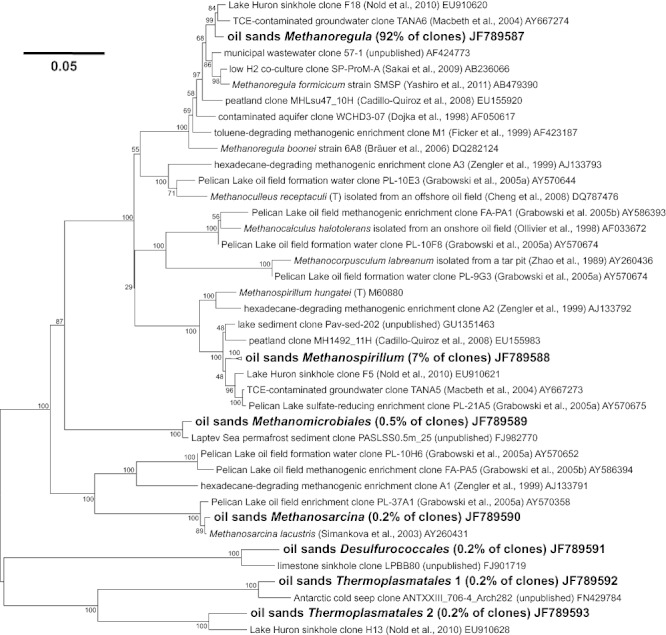
Neighbour joining phylogenetic tree indicating taxonomic affiliations of cloned archaeal 16S rRNA gene sequences from Athabasca oil sands formation waters. For each of the archaeal OTUs the most closely related sequences from GenBank and other related sequences of interest (e.g. from other petroleum reservoirs or methanogenic hydrocarbon-degrading systems) are indicated. Bootstrap values next to branching nodes are based on 100 resamplings.

Putative hydrogenotrophic methanogens from the order *Methanomicrobiales* accounted for 410 out of 414 archaeal sequences retrieved from the oil sands formation water, comprising two dominant phylotypes related to *Methanoregula* and *Methanospirillum* spp. (Table 2; Fig. 3). Closely related *Methanoregula* spp. (> 98%; Fig. 3) have been detected in lake bottom sinkholes (e.g. Nold *et al*., 2010), a TCE-contaminated aquifer (Macbeth *et al*., 2004), a toluene-degrading enrichment (Ficker *et al*., 1999) and peatlands (e.g. Cadillo-Quiroz *et al*., 2008). One such peat bog was the source for the isolation of the type species *Methanoregula boonei* (Bräuer *et al*., 2006), an H_2_- and CO_2_-utilizing acidophilic methanogen that does not use other substrates (e.g. acetate, formate or trimethylamine; Bräuer *et al*., 2011) and is the closest cultured relative to the dominant oil sands phylotype (Fig. 3). The second most abundant phylotype has the hydrogenotrophic *Methanospirillum hungatei* as its closest cultured relative. Closely related *Methanospirillum* spp. have also been detected in sinkholes, aquifers and peatlands (see Fig. 3), and also include a *Methanospirillum* sp. enriched from the Pelican Lake oil reservoir *c*. 200 km to the west of these oil sands (Grabowski *et al*., 2005a; Figs 1 and 3). One out of the 414 sequences retrieved was affiliated with the *Methanosarcinales* (Table 2; Fig. 3) and is closely related to a *Methanosarcina* sp. detected in an archaeal 16S rRNA gene clone library from Pelican Lake formation water (without prior selective enrichment). Three more singletons from the archaeal libraries were affiliated with non-methanogen sequences retrieved from cold seeps and sinkholes (Table 2; Fig. 3).

### *Bacteria* detected in oil sands formation waters

**Fig 4:**
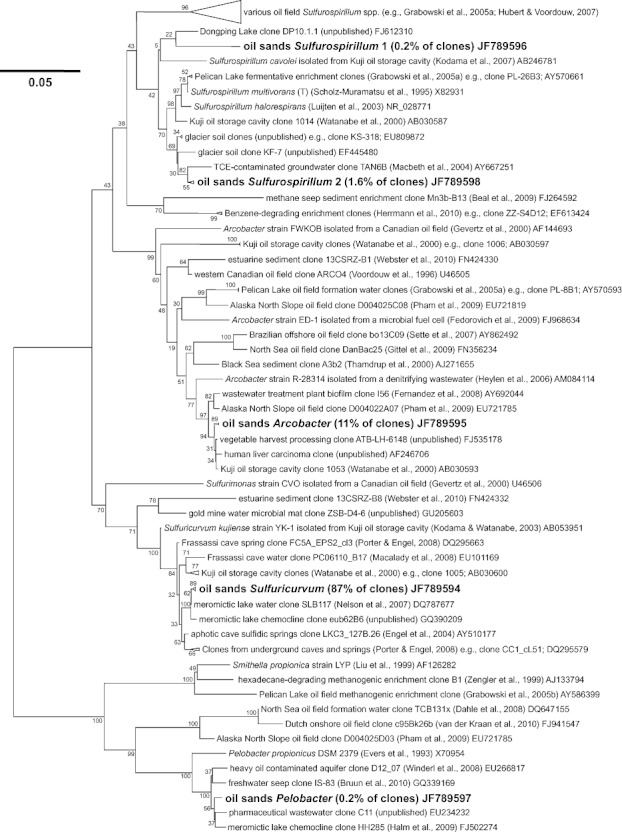
Neighbour joining phylogenetic tree indicating taxonomic affiliations of cloned bacterial 16S rRNA gene sequences from Athabasca oil sands formation waters. For each of the bacterial OTUs the most closely related sequences from GenBank and other related sequences of interest (e.g. from other petroleum reservoir habitats) are indicated. Bootstrap values next to branching nodes are based on 100 resamplings.

Bacterial sequences affiliated with *Sulfuricurvum* were most closely related to environmental clones derived from meromictic lake sediments (Nelson *et al*., 2007), chemoclines (unpublished GenBank entry Accession No. GQ390209; see Fig. 4), and sulfidic caves and springs (Porter and Engel, 2008) (Fig. 4). The only cultivated *Sulfuricurvum* reported so far are strains of *Sulfuricurvum kujiense* isolated from an underground crude oil storage cavity at Kuji, Japan (Kodama and Watanabe, 2003; 2004), which have high 16S rRNA sequence identity to the oil sands formation water *Sulfuricurvum* sp. 16S rRNA sequence (98%; Fig. 4). *Sulfuricurvum kujiense* strain YK-1 is considered an obligate chemolithotroph (Kodama and Watanabe, 2004) that can grow on crude oil by coupling nitrate reduction to the oxidation of reduced, presumably organic, sulfur compounds in petroleum (Kodama and Watanabe, 2003). *Sulfuricurvum kujiense* cannot use hydrocarbons directly as a carbon and energy source but is able to oxidize sulfide, elemental sulfur, thiosulfate and hydrogen (Kodama and Watanabe, 2003; 2004).

*Arcobacter* spp. are found in various habitats (Gevertz *et al*., 2000; Campbell *et al*., 2006; Miller *et al*, 2007; Fedorovich *et al*., 2009; Webster *et al*., 2010), and those closely related to the oil sands phylotype come from a range of environments (Fig. 4), including the Kuji oil cavity (Watanabe *et al*., 2000). The most closely related cultured *Arcobacter* (strain R-28314; Fig. 4) was isolated from a denitrifying wastewater treatment plant (Heylen *et al*., 2006). An *Arcobacter* that made up 100% of the bacterial 16S rRNA gene clone library in Pelican Lake formation waters (Grabowski *et al*., 2005a) shares 92% 16S rRNA sequence identity with the *Arcobacter* detected in the oil sands formation waters studied here (Fig. 4).

The two *Sulfurospirillum* phylotypes discovered in this study share 94% 16S rRNA sequence identity with each other (Fig. 4). *Sulfurospirillum* phylotype 2 has among its closest relatives an organism enriched from the Pelican Lake reservoir formation water in medium selective for fermentative bacteria (Grabowski *et al*., 2005a). Other relatives include uncultured bacteria from a TCE-contaminated subsurface aquifer (Macbeth *et al*., 2004) and soil associated with a Himalayan glacier (unpublished GenBank entries, e.g. Accession No. EU809872; Fig. 4). The closest cultivated relatives of both *Sulfurospirillum* phylotypes are *Sulfurospirillum multivorans* and *S. halorespirans*, which are metabolically versatile facultative chemolithotrophs (Luijten *et al*., 2003; Fig. 4). *Sulfurospirillum* phylotype 1 is most closely related (94%) to an uncultured lacustrine organism (unpublished GenBank entry Accession No. FJ612310; Fig. 4). *Sulfurospirillum* spp. have been detected in different oil field habitats, including the Kuji oil cavity, Pelican Lake and other fields in western Canada (Grabowski *et al*., 2005a; Hubert and Voordouw, 2007; Kodama *et al*., 2007; Fig. 4).

The *Pelobacter* sequence detected in the larger bacterial clone libraries (Table 2) is most closely related to uncultured *Deltaproteobacteria* from different environments including the sulfidic zone of an anoxic aquifer contaminated with heavy oil (Winderl *et al*., 2008). The most closely related described strain is *Pelobacter propionicus* strain DSM 2379 (Fig. 4), which ferments alcohols to organic acids (Schink *et al*., 1987).

### Intact polar lipids

Microbial community analysis of the formation water samples also included isolation and characterization of IPLs from biomass that was filtered from the three formation water samples. Phosphatidylethanolamines (PE) and phosphatidylglycerols (PG) were detected in all three samples (Table 1; for further details see Oldenburg *et al*., 2009), indicating that formation waters retrieved from distant dewatering wells (kilometres apart; Fig. 1) may harbour similar bacterial communities. IPL structures can be used to make taxonomic inferences (Sturt *et al*., 2004), and while this approach lacks the phylogenetic resolution of 16S rRNA-based comparisons, bulk IPL analyses are not prone to issues such as primer specificity that can result in selective amplification of 16S rRNA genes from certain taxa. PE and PG are not highly diagnostic, as they are characteristic of many bacteria including *Proteobacteria* (Dowhan, 1997). In a recent study PE and PG were reported to be the most abundant IPLs in the anoxic zone of the Black Sea water column (Schubotz *et al*., 2009) where nucleic acid-based cloning and fluorescence *in situ* hybridization analyses both revealed dominance of *Epsilonproteobacteria* (Vetriani *et al*., 2003; Lin *et al*., 2006), and high rates of sulfide oxidation are coupled to nitrate reduction (Jørgensen *et al*., 1991; Wakeham *et al*., 2007). We did not detect IPLs characteristic of archaea in oil sands formation waters, suggesting that bacteria are more abundant than archaea in these samples.

### Sulfur biogeochemistry in oil sands reservoir formation waters

During sampling of the reservoir formation waters, a distinct smell of hydrogen sulfide was noticed. The hydrogen sulfide concentration in the formation water samples was not measured during field sampling, but later chemical analysis detected up to 0.3 mM sulfate in the water samples (Table 1). Sulfur isotope analysis revealed mean δ^34^S values for elemental sulfur and sulfate of +26.7‰ and +23.3‰, respectively (Table 1), suggesting sulfate generation via microbial oxidation of elemental sulfur and possibly other reduced sulfur compounds. This 4‰ difference in δ^34^S between sulfur and sulfate is consistent with isotope fractionation effects observed in a pure culture of sulfide-oxidizing *Epsilonproteobacteria* (*Sulfurimonas* sp. strain CVO) from a western Canadian oil field, grown under nitrate-reducing conditions (Hubert *et al*., 2009).

**Fig 5:**
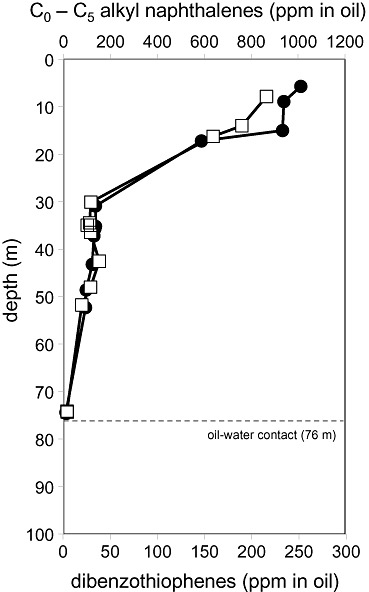
Concentrations of dibenzothiophenes (the sum of C_0–2_-alkyl dibenzothiophenes) in crude oil (white squares) and of C_0–5_-alkyl naphthalenes in crude oil (black circles) as a function of reservoir depth in Athabasca oil sands at the Muskeg River mine. The profiles are indicative of microbial degradation of crude oil compounds catalysed at the oil water transition zone over geological timescales (Head *et al*., 2003). Formation waters were obtained from dewatering wells penetrating the basal water leg at *c*. 80 m depth.

To assess the possibility that organic sulfur compounds provide electron donors for oil-associated *Epsilonproteobacteria* (as reported for *S. kujiense* strain YK-1; Kodama and Watanabe, 2003) dibenzothiophene concentrations were measured at different depths in drill core samples obtained from the oil sands layer overlying the formation waters. Figure 5 shows the dibenzothiophene concentration in oil decreasing from 215 ppm near the surface to 4 ppm near the oil–water contact at 80 m. This profile mirrors the general trends of oil biodegradation with depth, as shown by the concentrations of C_0_ to C_5_ alkyl naphthalenes, which decrease from 1011 ppm to 12 ppm in the same samples (alkyl aromatic hydrocarbon distributions and concentrations are indicative of the extent of biodegradation in heavy oils; Head *et al*., 2003; Larter *et al*., 2008). These depth profiles are consistent with microbial activity occurring over geological timescales at the oil water contact (*c*. 80 m depth in this system; further details provided in Fustic *et al*., 2011), and suggest that microbial desulfurization of crude oil compounds has occurred in these oil sands.

## Discussion

**Fig 6:**
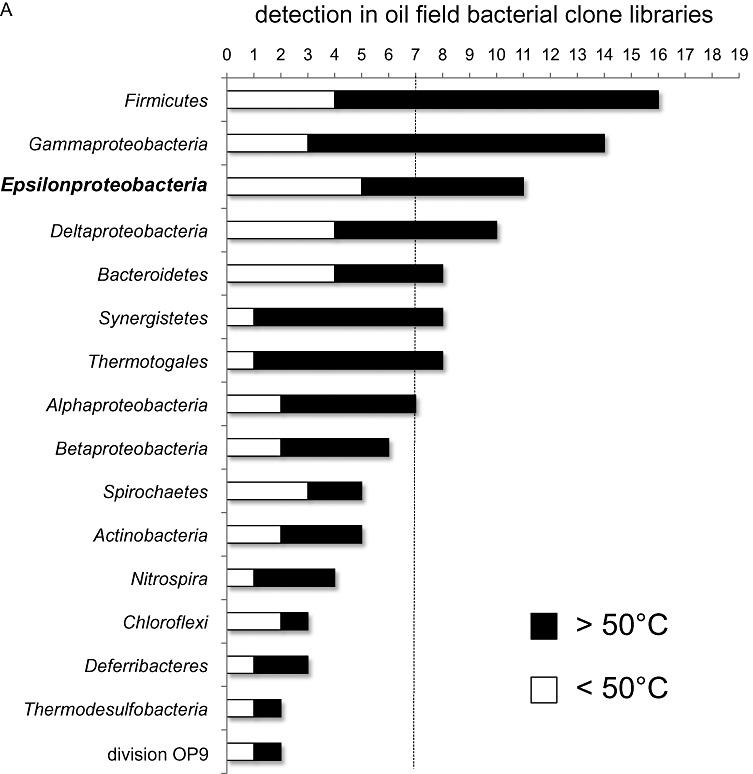

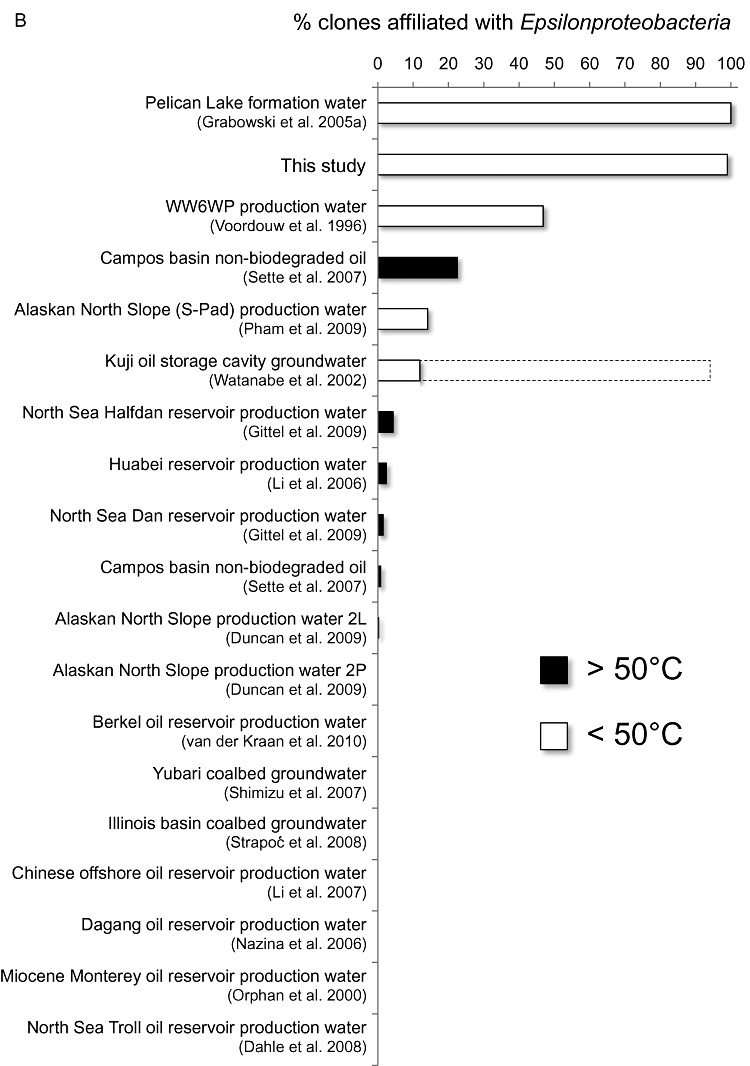
Results from 19 published bacterial clone libraries (references are given in B) from subsurface fossil fuel reservoirs were evaluated for the occurrence of dominant taxa. Seven of the habitats were low-temperature environments (reported as < 50°C *in situ*) as indicated by the dashed line in (A), and 12 were high-temperature oil reservoirs (> 50°C). (A) shows that *Epsilonproteobacteria* are the third most frequently occurring group overall and were detected in five out of seven low-temperature systems. The abundance of *Epsilonproteobacteria* in these oil field clone libraries is summarized in (B), which indicates that *Epsilonproteobacteria* are particularly abundant in low-temperature oil fields in western Canada. The hatched bar indicates the discrepancy in results reported from the Kuji oil storage cavity (Watanabe *et al*., 2000; 2002) as described in the *Introduction*.

An analysis of 19 clone libraries of bacterial 16S rRNA genes from different oil fields revealed that *Epsilonprotoeobacteria* are among the most frequently detected taxa in fossil fuel reservoir production fluids (Fig. 6). On the basis of these published reports, only *Firmicutes* and *Gammaproteobacteria* have been detected in oil fields more often than *Epsilonproteobacteria*. Grouping these 19 studies according to low-temperature (< 50°C) and high-temperature (> 50°C) reservoirs reveals that in low-temperature systems like the near-surface oil sands reservoir investigated here, *Epsilonprotoeobacteria* are the most frequently reported bacterial group (Fig. 6A). Furthermore, in low-temperature oil fields in western Canada, *Epsilonprotoeobacteria* are represented at high abundance in 16S rRNA gene clone libraries (Fig. 6B), as demonstrated here for the Athabasca oil sands (Table 2). By applying multiple bacterial PCR primers to DNA extracted using different techniques, our study confirms that *Epsilonproteobacteria* are indigenous to low-temperature petroleum reservoirs where they are abundant members of the microbial community; their massive dominance inferred from several 16S rRNA gene-based analyses is unlikely to be the result of primer selectivity as has been previously suggested (Grabowski *et al*., 2005a; Pham *et al*., 2009; Head *et al*., 2010). The oil sands at the Muskeg River mine are part of the same Athabasca heavy oil system as the Pelican Lake reservoir (Fig. 1). The recovery of 16S rRNA gene sequences from *Epsilonproteobacteria* (99–100% of clones) in pristine formation waters from both oil fields is in good agreement, and suggests that the dominance of *Arcobacter* spp. in the Pelican Lake formation waters (Grabowski *et al*., 2005a) reflects an important role or roles for *Epsilonproteobacteria* in heavy oil reservoirs.

The most abundant phylotype in all three of our bacterial libraries belonged to the genus *Sulfuricurvum*. Unlike the other epsilonproteobacterial genera detected (*Arcobacter* and *Sulfurospirillum* spp.; Table 2; Fig. 4), *Sulfuricurvum* spp. are not known to be capable of chemoorganotrophic metabolism on the basis of substrate tests with the only cultivated member of this genus, *S. kujiense* strain YK-1 (Kodama and Watanabe, 2003; 2004). Despite being autotrophic, strain YK-1 grows particularly well by oxidizing reduced, most probably organic, sulfur compounds in crude oil. This trait may explain *Sulfuricurvum* dominating an oil sands environment with a relatively high sulfur content (Strausz and Lown, 2003). The oil–water contact in the Muskeg River oil sands shows distinct gradients of dibenzothiophenes indicative of biodegradation in the aquifer associated with the oil sands (Fig. 5; Head *et al*., 2003). Biological sulfur oxidation in these oil sands is apparent from the isotopic composition of sulfate (mean +23.3‰) being enriched in ^32^S relative to elemental sulfur (mean +26.7‰) in the formation waters, even in the absence of nitrate (Table 1) – the electron acceptor that supports growth of *S. kujiense* on organic sulfur compounds in oil (Kodama and Watanabe, 2003). Oxidation of reduced sulfur compounds *in situ* may have been driven by microbial reduction of metal species (Aller and Rude, 1988; Beal *et al*., 2009), and the presence of Fe^2+^ and Mn^2+^ in the formation waters (Table 1) is indicative of metal cycling in this system. Many *Arcobacter* and *Sulfurospirillum* spp. have been physiologically characterized and some are capable of iron or manganese reduction (Thamdrup *et al*., 2000; Fedorovich *et al*., 2009; Fry *et al*., 2009); however, *S. kujiense* strain YK-1 has not been tested for this capability. Like some other *Epsilonproteobacteria*, strain YK-1 can grow under microaerophilic conditions (Kodama and Watanabe, 2003; 2004), and although these oil sands formation waters were noticeably sulfidic during sampling, rapid metabolism of low levels of oxygen entering this system via groundwater flow cannot be excluded.

*Sulfuricurvum* spp. have been detected in many groundwater environments (Campbell *et al*., 2006; Porter and Engel, 2008; Fig. 4). The Kuji underground crude oil storage cavity (the source of strain YK-1; Kodama and Watanabe, 2004) is not a pristine oil reservoir like the oil sands studied here, but rather an engineered subsurface habitat where temporarily stored crude oil comes into contact with the local groundwater (Watanabe *et al*., 2000). Indeed, *Sulfuricurvum* spp. have not been reported in oil field microbial diversity studies (e.g. those indicated in Fig. 6) until now. The subterranean biogeography of several *Sulfuricurvum* spp. in groundwaters (Porter and Engel, 2008) suggests that a physical connection may exist between this Athabasca oil sands reservoir and the surrounding basal aquifer, which would influence the oil sands reservoir microbiota and biogeochemistry at oil water contact zones where biodegradation occurs (Head *et al*., 2003). The ways in which indigenous petroleum reservoir microbial communities establish are largely unknown, and groundwater infiltration – thought to influence microbial community composition in some coal deposits (Tseng *et al*., 1998; Schlegel *et al*., 2011) – may influence the resident microbiota in shallow western Canadian biodegraded oil sands.

Hydrogenotrophic methanogenesis is thought to be responsible for the formation of heavy oil (Jones *et al*., 2008), and depends on CO_2_ and H_2_ production via fermentation of crude oil components. It remains unclear which taxa catalyse syntrophic fermentations in biodegraded subsurface petroleum reservoir environments (Pham *et al*., 2009), and the predominance of *Epsilonproteobacteria* is intriguing in this context. Certain studies suggest *Epsilonproteobacteria* might be involved in syntrophic anaerobic communities that degrade hydrocarbons. For example, an epsilonproteobacterium was recently shown to assimilate ^13^C during syntrophic degradation of ^13^C-labelled benzene in sulfate-reducing enrichment cultures (Herrmann *et al*., 2010) and appears to contribute to benzene fermentation, e.g. into H_2_, acetate and CO_2_. Indeed in the study of Herrmann and colleagues (2010), the highest ^13^C enrichment was observed for the *Epsilonproteobacteria*. *Sulfurospirillum* spp. can ferment a wide variety of organic compounds (Luijten *et al*., 2003) and were among the fermentative heterotrophs isolated from a subsurface coal deposit (Fry *et al*., 2009) and the Pelican Lake oil reservoir (Grabowski *et al*., 2005a; Fig. 4). Acetate is metabolized by *Arcobacter* spp. in various anoxic settings (Thamdrup *et al*., 2000; Fedorovich *et al*., 2009; Webster *et al*., 2010). Syntrophic oxidation of acetate to H_2_ and CO_2_ is important in some methanogenic crude oil degrading systems (Jones *et al*., 2008), and is catalysed by a few cultured isolates (Hattori, 2008; Westerholm *et al*., 2010) that can also catalyse the reverse reaction (i.e. acetogenesis from H_2_ and CO_2_; Lee and Zinder, 1988). Several *Epsilonproteobacteria* can use H_2_ as an electron donor (e.g. Gevertz *et al*., 2000; Kodama *et al*., 2007), including the acetogenic *Arcobacter* dominating the bacterial community in the Pelican Lake reservoir (Grabowski *et al*., 2005a). This is consistent with *Arcobacter* sequences dominating the metagenome of formation water from a coalbed methane reservoir (US Patent 2010/0047793 A1, 2010), a habitat where syntrophic methanogenic hydrocarbon degradation also occurs (Strąpoć*et al*., 2008).

Intact polar lipid analyses indicated that bacteria predominated over archaea in the formation waters (Oldenburg *et al*., 2009). Taken together, lipid and DNA-based results therefore suggest a *Sulfuricurvum* sp. is the most abundant organism in this heavily biodegraded oil reservoir, with *Arcobacter*, *Sulfurospirillum* and hydrogenotrophic *Methanomicrobiaceae* spp. making up smaller fractions of the microbial community (Table 2). The latter groups may be involved in methanogenic crude oil degradation that has generated these and other heavy oils, and currently these populations may have given way to a dibenzothiophene-degrading *Sulfuricurvum* population well suited to this sulfur-enriched oil sands reservoir (around 5% sulfur by weight; Marcano, 2011). Our results provide clear evidence that 16S rRNA gene-based detection of *Epsilonproteobacteria* in oil field formation waters is not artefactual. On the contrary, *Epsilonproteobacteria* are predominant members of low-temperature biodegraded petroleum reservoirs, and their ecophysiology in such environments can be explained by examining different aspects of crude oil sulfur and hydrocarbon biogeochemistry.

## Experimental procedures

### Sample collection

Athabasca oil sands formation waters were sampled at the Muskeg River oil sands mine north of Fort McMurray, Alberta, Canada (Fig. 1). Mining operations at this site require reservoir formation water discharge in advance of open pit oil sands removal and water from three out of the six dewatering wells surrounding the active excavation area were sampled (Fig. 1). Wellhead valves were opened and flushed for several minutes prior to collecting 12 or 24 l using a sterilized metal funnel to completely fill sterile 4 l brown glass bottles that had previously been flushed with nitrogen gas. Water samples were transported to the laboratory in a cool box where they were subsequently stored at 4°C. Oil sands samples were obtained from areas of the reservoir that had been undergoing commercial excavation. Large volumes (several m^3^) of sediment were excavated from the intact formation by mine equipment (large backhoe). This enabled aseptic subsampling from the middle part of the excavated oil sands. Subsamples were stored anaerobically in N_2_-flushed containers kept at room temperature. Oil sands were also obtained from vertical drill cores spanning the oil leg and reaching depths close to the oil water transition zone.

### Geochemical analysis

Formation water samples from dewatering wells numbered 1, 4 and 6 were subject to a suite of geochemical analyses, performed by the Analytical Geochemistry Group at the University of Calgary. To determine isotopic compositions of sulfate and elemental sulfur in the formation waters, BaSO_4_ was precipitated following addition of 0.5 M BaCl_2_ and elemental sulfur was extracted following filtration and Bligh and Dyer extraction (Bligh and Dyer, 1959). Sulfur isotope ratios were determined by continuous flow-elemental analysis-isotope ratio mass spectrometry (CF-EA-IRMS) with a Finnigan Mat Delta+XL spectrometer interfaced with a Carlo Erba NA 1500 elemental analyser and are expressed relative to the international standard Canyon Diablo Troilite. For IPL analysis, biomass was concentrated by vacuum filtration of the 12 or 24 l samples through 0.2 µm pore size filters (Millipore) that were subsequently freeze dried to allow biomass removal by scraping it from the filters with a sterilized metal spatula. The biomass was then subjected to a modified Bligh and Dyer extraction (Bligh and Dyer, 1959) to recover polar fractions which were analysed by liquid chromatography-mass spectrometry to determine IPL composition, as described previously (Oldenburg *et al*., 2009).

Bitumen samples from the oil sands drill core were subjected to petroleum geochemistry analyses. ‘Aromatic hydrocarbon’ fractions including dibenzothiophenes were isolated from de-asphaltened bitumen extracts following the procedure of Bennett and Larter (2000). Alkyl naphthalenes were quantified relative to a D8-naphthalene standard (response factors of unity were employed therefore data can be considered semi-quantitative). Mass spectral characterization of compounds in the aromatic hydrocarbon fractions was carried out using splitless injection GC MS with an Agilent 6890 chromatograph interfaced to a 5973 quadrupole mass-selective detector. Saturated and aromatic hydrocarbons were analysed with a DB-5-coated fused silica column (30 m length, 0.32 mm id, 0.25 µm film thickness; J&W Scientific) according to the following temperature programme: 40°C (held for 2 min) to 300°C (held for 20 min) increasing at 4°C min^−1^.

### DNA extraction and PCR amplification of 16S rRNA genes

Most of the biomass isolated by filtration was dedicated to IPL analysis as described above. DNA was extracted from the remaining biomass residue on the same filters, which allowed for 16S rRNA gene analyses. Initial screening was performed by DGGE to confirm that the microbial communities in the three water samples were similar. Subsequent community analyses were performed on water from wellhead #1 according to two complementary approaches:

(i) At the University of Calgary, filters were submerged in a resuspension buffer (0.15 M NaCl; 0.1 M EDTA) and incubated on a rotary shaker overnight. DNA was extracted from resuspended biomass using the method of Marmur (Marmur, 1961) with modifications (Voordouw *et al*., 1990) including successive rounds of freezing and thawing as well as lysozyme, RNase and proteinase K treatments. DNA concentrations were measured by spotting dilutions on a square of 1% agarose gel containing ethidium bromide (*c*. 10 µg ml^−1^). The concentration of DNA was estimated under UV illumination by comparison with fluorescence of known amounts of phage lambda DNA. The presence of bacterial DNA was confirmed in all formation water samples by positive PCR amplification from all DNA extracts using primers 8f and 1406r (see Table S1). Extracted DNA from wellhead #1 formation water was sent to the JCVI in Rockville for amplification of 16S rRNA genes from *Archaea* using primers arch8f and arch1492r, and from *Bacteria* using primers 9f and 1545r (Table S1). Amplifications were performed using a DNA Engine Tetrad PTC-225 thermal cycler (MJ Research, Waltham, MA) with an initial denaturation of 2 min at 94°C, followed by 29 cycles of 30 s at 94°C, 30 s at 55°C and 2 min at 72°C, with a final extension of 5 min at 72°C. A negative control PCR reaction in which the genomic DNA template was replaced by an equivalent volume of sterile distilled water was also included.
(ii) Parallel analysis was conducted at Newcastle University where DNA was extracted directly from the filters for each wellhead formation water sample using the FastDNA Spin Kit for soil (Q-BIOgene, UK). Filters containing biomass residue were cut into small sections and added to 2 ml tubes and DNA was extracted following the manufacturer's guidelines, which include an initial bead-beating step using a RiboLyzer (Hybaid). 16S rRNA genes from *Archaea* were amplified by PCR using primers arch46f and arch1017r. 16S rRNA genes from *Bacteria* were amplified using two different primer pairs, either 8f and 1542r or inosine-341f and 1492r (Table 2). All primer sequences and a summary of downstream procedures used to analyse the amplified 16S rRNA genes are listed in Table S1.

### Denaturing gradient gel electrophoresis (DGGE)

Shorter 16S rRNA gene fragments were amplified for DGGE. For bacterial 16S rRNA genes a PCR product generated using primers 341f-GC and 534r was used directly for DGGE. Bacterial DGGE was also performed following nested PCR amplifications where PCR products generated with inosine-341f and 1492r or 8f and 1542r were used as templates for a second PCR reaction with 341f-GC and 534r. A nested approach was also used to analyse archaeal 16S rRNA gene fragments by DGGE. PCR products generated with primers arch46f and arch1017r were used as template for a second round of PCR using primers arch344f-GC and Uni522r. All primer sequences are listed in Table S1. DGGE was conducted in 10% acrylamide gels with 30–60% denaturant, as described previously (Rowan *et al*., 2003). Acrylamide gels were run for 4 h at 200 volts using the D gene system (Bio-Rad, Hemel Hempstead, UK) and were subsequently stained for 30 min in SYBR green I (Sigma, Poole, UK; diluted 1/10000 in 1× TAE). Stained gels were viewed under ultraviolet light and gel images were recorded using a Bio-Rad Fluor-S® MultiImager (Bio-Rad, UK). Bands of interest were excised for sequencing.

### Cloning, sequencing and phylogenetic analyses

Bacterial and archaeal 16S rRNA gene fragments were cloned using TOPO TA cloning kits (Invitrogen) according to the manufacturer's instructions. Approximately 384 clones were analysed from the JCVI libraries, and approximately 96 clones were analysed from each of the Newcastle libraries. Nearly complete full-length 16S rRNA gene sequences (*Escherichia coli* positions 9–1545) were obtained from the JCVI libraries by sequencing using M13 cloning vector primers. Partial 16S rRNA gene sequences were obtained from Newcastle clones using internal 16S rRNA gene primers (Edwards *et al*., 1989). Closely related sequences from GenBank were identified by blast searching (Altschul *et al*., 1990) of the GenBank database and using the Ribosomal Database Project SeqMatch tool (Cole *et al*., 2007). Sequences were aligned using the SILVA web aligner (Pruesse *et al*., 2007) and alignments were manually corrected using BioEdit (Hall, 1999). Neighbour joining phylogenetic trees were constructed using MEGA4 (Tamura *et al*., 2007). Sequence alignments for phylogenetic tree reconstruction included the top three hits obtained in blast and SeqMatch searches (multiple environmental sequences from the same study or habitat were considered as one hit) to each of the oil sands phylotypes, as well as other sequences of interest. Representative sequences from the formation water clone libraries have been deposited in the GenBank database under Accession No. JF789587 to JF789598.

### Meta-analysis of published oil field 16S rRNA bacterial clone library results

A literature survey revealed 19 published clone libraries of bacterial 16S rRNA genes from oil reservoir production fluids. Clone library results were collated to reveal the proportion of clones affiliated with major bacterial divisions. This analysis considered over 3600 sequences of cloned 16S rRNA genes, which we classified at the level of major phylogenetic groups (phylum/subphylum level). From these data, distributions based on the frequency of occurrence and average percentage representation of major taxa in clone libraries could be plotted (see Fig. 6). Only clone libraries that used broad specificity (‘universal’) primers for PCR amplification of 16S rRNA genes were included in the survey. Published DGGE analyses on oil field production fluid samples were not included in this analysis. Results were considered in the context of low- and high-temperature petroleum reservoirs using a cut-off of 50°C (*in situ* reservoir temperature as indicated in the publications). We recently used a similar approach to conduct a broad assessment of microbial community structure in petroleum-impacted environments including soils and surface sediments (Gray *et al*., 2010).

## Supporting information

Additional Supporting Information may be found in the online version of this article: **Table S1.** PCR primers used for microbial community analyses. Please note: Wiley-Blackwell are not responsible for the content or functionality of any supporting materials supplied by the authors. Any queries (other than missing material) should be directed to the corresponding author for the article.

## References

[b1] Aller RC, Rude PD (1988). Complete oxidation of solid phase sulfides by manganese and bacteria in anoxic marine sediments. Geochim Cosmochim Acta.

[b2] Altschul SF, Gish W, Miller W, Myers EW, Lipman DJ (1990). Basic local alignment search tool. J Mol Biol.

[b120] Beal EJ, House CH, Orphan VJ (2009). Manganese- and Iron-Dependent Marine Methane Oxidation. Science.

[b3] Bennett B, Larter SR (2000). Quantitative separation of aliphatic and aromatic hydrocarbons using silver ion–silica solid-phase extraction. Anal Chem.

[b4] Bligh EG, Dyer WJ (1959). A rapid method of total lipid extraction and purification. Can J Biochem Physiol.

[b5] Bräuer SL, Cadillo-Quiroz H, Yashiro E, Yavitt JB, Zinder SH (2006). Isolation of a novel acidiphilic methanogen from an acidic peat bog. Nature.

[b6] Bräuer SL, Cadillo-Quiroz H, Ward RJ, Yavitt JB, Zinder SH (2011). *Methanoregula boonei* gen. nov., sp. nov., an acidiphilic methanogen isolated from an acidic peat bog. Int J Syst Appl Microbiol.

[b24] Bruun A-M, Finster K, Gunnlaugsson HP, Nornberg P, Friedrich MW (2010). A Comprehensive Investigation on Iron Cycling in a Freshwater Seep Including Microscopy, Cultivation and Molecular Community Analysis. Geomicrobiol J.

[b7] Cadillo-Quiroz H, Yashiro E, Yavitt JB, Zinder SH (2008). Characterization of the archaeal community in a minerotrophic fen and terminal restriction fragment length polymorphism-directed isolation of a novel hydrogenotrophic methanogen. Appl Environ Microbiol.

[b8] Campbell BJ, Engel AS, Porter ML, Takai K (2006). The versatile *Epsilonproteobacteria*: key players in sulphidic habitats. Nat Rev Microbiol.

[b27] Cheng L, Qiu TL, Li X, Wang WD, Deng Y, Yin XB, Zhang H (2008). Isolation and characterization of Methanoculleus receptaculi sp. nov. from Shengli oil field, China. FEMS Microbiol Lett.

[b9] Cole JR, Chai B, Farris RJ, Wang Q, Kulam-Syed-Mohideen AS, McGarrell DM (2007). The ribosomal database project (RDP-II): introducing *myRDP* space and quality controlled public data. Nucleic Acid Res.

[b29] Dahle H, Garshol F, Madsen M, Birkeland NK (2008). Microbial community structure analysis of produced water from a high-temperature North Sea oil-field. Antonie Van Leeuwenhoek.

[b30] Dojka MA, Hugenholtz P, Haack SK, Pace NR (1998). Microbial diversity in a hydrocarbon- and chlorinated-solvent-contaminated aquifer undergoing intrinsic bioremediation. Appl Environ Microbiol.

[b12] Dowhan W (1997). Molecular basis for membrane phospholipid diversity: why are there so many lipids?. Annu Rev Biochem.

[b32] Duncan KE, Gieg LM, Parisi VA, Tanner RS, Greene Tringhe S, Bristow J, Suflita JM (2009). Biocorrosive thermophilic microbial communities in Alaskan north slope oil facilities. Environ Sci Technol.

[b04] Edwards U, Rogall T, Blöcker H, Emde M, Böttger EC (1989). Isolation and direct complete nucleotide determination of entire genes. Characterisation of a gene coding for 16S ribosomal RNA. Nucl Acids Res.

[b34] Engel AS, Porter ML, Stern LA, Quinlan S, Bennett PC (2004). Bacterial diversity and ecosystem function of filamentous microbial mats from aphotic (cave) sulfidic springs dominated by chemolithoautotrophic “Epsilonproteobacteria”. FEMS Microbiol Ecol.

[b14] ERCB (Alberta Energy Resources Conservation Board) (2010).

[b36] Evers S, Weizenegger M, Ludwig W, Schink B, Schleifer KH (1993). The phylogenetic positions of Pelobacter acetylenicus and Pelobacter propionicus. Syst Appl Microbiol.

[b15] Fedorovich V, Knighton MC, Pagaling E, Ward FB, Free A, Goryanin I (2009). Novel electrochemically active bacterium phylogenetically related to *Arcobacter butzleri*, isolated from a microbial fuel cell. Appl Environ Microbiol.

[b38] Fernandez N, Diaz EE, Amils R, Sanz JL (2008). Analysis of Microbial Community during Biofilm Development in an Anaerobic Wastewater Treatment Reactor. Mol Ecol.

[b16] Ficker M, Krastel K, Orlicky S, Edwards E (1999). Molecular characterization of a toluene-degrading methanogenic consortium. Appl Environ Microbiol.

[b17] Fry JC, Horsfield B, Sykes R, Cragg BA, Heywood C, Kim GT (2009). Prokaryotic populations and activities in an interbedded coal deposit, including a previously deeply buried section (1.6–2.3 km) above ∼150 Ma basement rock. Geomicrobiol J.

[b18] Fustic M, Bennett B, Huang H, Oldenburg T, Hubbard S, Larter S, Hein FJ, Suter J, Leckie DA, Larter S (2011). Impact of oil–water contacts, reservoir (dis)continuity, and reservoir characteristics on spatial distribution of water, gas, and high-water – low-bitumen saturated zones and variability of bitumen properties in Athabasca oil sands deposits. Heavy Oil/Bitumen Petroleum Systems in Alberta & Beyond.

[b19] Gevertz D, Telang AJ, Voordouw G, Jenneman GE (2000). Isolation and characterization of strains CVO and FWKO B, two novel nitrate-reducing, sulfide-oxidizing bacteria isolated from oil field brine. Appl Environ Microbiol.

[b21] Gittel A, Sørensen KB, Skovhus TL, Ingvorsen K, Schramm A (2009). Prokaryotic community structure and sulfate reducer activity in water from high-temperature oil reservoirs with and without nitrate treatment. Appl Environ Microbiol.

[b22] Grabowski A, Nercessian O, Fayolle F, Blanchet D, Jeanthon C (2005a). Microbial diversity in production waters of a low-temperature biodegraded oil reservoir. FEMS Microbiol Ecol.

[b23] Grabowski A, Blanchet D, Jeanthon C (2005b). Characterization of long-chain fatty-acid-degrading syntrophic associations from a biodegraded oil reservoir. Res Microbiol.

[b121] Gray ND, Sherry A, Larter SR, Erdmann M, Leyris J, Liengen T (2009). Biogenic methane production in formation waters from a large gas field in the North Sea. Extremophiles.

[b25] Gray ND, Sherry A, Hubert C, Dolfing J, Head IM (2010). Methanogenic degradation of petroleum hydrocarbons in subsurface environments: remediation, heavy oil formation, and energy recovery. Adv Appl Microbiol.

[b26] Grigoryan A, Voordouw G (2008). Microbiology to help solve our energy needs methanogenesis from oil and the impact of nitrate on the oil-field sulfur cycle. Ann N Y Acad Sci.

[b122] Hall TA (1999). BioEdit: a user-friendly biological sequence alignment editor and analysis program for Windows 95/98/NT. Nucleic Acids Symp Ser.

[b50] Halm H, Musat N, Lam P, Langlois R, Musat F, Peduzzi S (2009). Co-occurrence of denitrification and nitrogen fixation in a meromictic lake, Lake Cadagno (Switzerland). Environ Microbiol.

[b28] Hattori S (2008). Syntrophic acetate-oxidizing microbes in methanogenic environments. Microbes Environ.

[b123] Head IM, Jones MD, Larter SL (2003). Biological activity in the deep subsurface and the origin of heavy oil. Nature.

[b124] Head IM, Larter SR, Gray ND, Sherry A, Adams JJ, Aitken CM, Timmis KN, McGenity T, van der Meer JR, de Lorenzo V (2010). Hydrocarbon degradation in petroleum reservoirs. Handbook of Hydrocarbon and Lipid Microbiology.

[b125] Herrmann S, Kleinsteuber S, Chatzinotas A, Kuppardt S, Lueders T, Richnow HH, Vogt C (2010). Functional characterization of an anaerobic benzene-degrading enrichment culture by DNA stable isotope probing. Environ Microbiol.

[b33] Heylen K, Vanparys B, Wittebolle L, Verstraete W, Boon N, de Vos P (2006). Cultivation of denitrifying bacteria: optimization of isolation conditions and diversity study. Appl Environ Microbiol.

[b35] Hubert C, Voordouw G (2007). Oil field souring control by nitrate-reducing *Sulfurospirillum* spp. that outcompete sulfate-reducing bacteria for organic electron donors. Appl Environ Microbiol.

[b126] Hubert C, Voordouw G, Mayer B (2009). Elucidating microbial processes in nitrate- and sulfate-reducing systems using sulfur and oxygen isotope ratios: the example of oil reservoir souring control. Geochim Cosmochim Acta.

[b127] Jeanthon C, Nercessian I, Corre E, Grabowski-Lux A, Ollivier B, Magot M (2005). Hyperthermophilic and methanogenic Archaea. Petroleum Microbiology.

[b39] Jones DM, Head IM, Gray ND, Adams JJ, Rowan AK, Aitken CM (2008). Crude-oil biodegradation via methanogenesis in subsurface petroleum reservoirs. Nature.

[b40] Jørgensen BB, Fossing H, Wirsen CO, Jannasch HW (1991). Sulfide oxidation in the anoxic Black Sea chemocline. Deep Sea Res.

[b41] Kodama Y, Watanabe K (2003). Isolation and characterization of a sulfur-oxidizing chemolithotroph growing on crude oil under anaerobic conditions. Appl Environ Microbiol.

[b42] Kodama Y, Watanabe K (2004). *Sulfuricurvum kujiense* gen. nov., sp. nov., a facultatively anaerobic, chemolithoautotrophic, sulfur-oxidizing bacterium isolated from an underground crude-oil storage cavity. Int J Syst Evol Microbiol.

[b43] Kodama Y, Ha LT, Watanabe K (2007). *Sulfurospirillum cavolei* sp. nov., a facultatively anaerobic sulfur-reducing bacterium isolated from an underground crude oil storage cavity. Int J Syst Evol Microbiol.

[b64] van der Kraan GM, Bruining J, Lomans BP, van Loosdrecht MCM, Muyzer G (2010). Microbial diversity of an oil–water processing site and its associated oil field: the possible role of microorganisms as information carriers from oil-associated environments. FEMS Microbiol Ecol.

[b46] Larter S, Wilhelms A, Head I, Koopmans M, Aplin A, Di Primio R (2003). The controls on the composition of biodegraded oils in the deep subsurface – Part 1: Biodegradation rates in petroleum reservoirs. Org Geochem.

[b44] Larter S, Huang H, Adams J, Bennett B, Jokanola O, Oldenburg T (2006). The controls on the composition of biodegraded oils in the deep subsurface: Part II – Geological controls on subsurface biodegradation fluxes and constraints on reservoir-fluid property prediction. Am Assoc Petrol Geol Bull.

[b45] Larter S, Adams J, Gates ID, Bennett B, Huang H (2008). The origin, prediction and impact of oil viscosity heterogeneity on the production characteristics of tar sand and heavy oil reservoirs. J Can Petrol Technol.

[b47] Lee MJ, Zinder SH (1988). Isolation and characterization of a thermophilic bacterium which oxidizes acetate in syntrophic association with a methanogen and which grows acetogenically on H_2_-CO_2_. Appl Environ Microbiol.

[b69] Li H, Yang SZ, Mu BZ, Rong ZF, Zhang J (2006). Molecular analysis of the bacterial community in a continental high-temperature and water-flooded petroleum reservoir. FEMS Microbiol Lett.

[b70] Li H, Yang SZ, Mu BZ, Rong ZF, Zhang J (2007). Molecular phylogenetic diversity of the microbial community associated with a high-temperature petroleum reservoir at an offshore oilfield. FEMS Microbiol Ecol.

[b129] Lin X, Wakeham SG, Putnam IF, Astor YM, Scranton MI, Chistoserdov AY, Taylor GT (2006). Vertical distributions of prokaryotic assemblages in the anoxic Cariaco Basin and Black Sea compared using fluorescence *in situ* hybridization (FISH) techniques. Appl Environ Microbiol.

[b72] Liu Y, Balkwill DL, Aldrich HC, Drake GR, Boone DR (1999). Characterization of the anaerobic propionate-degrading syntrophs Smithella propionica gen. nov., sp. nov. and Syntrophobacter wolinii. Int J Syst Bacteriol.

[b51] Luijten MLGC, de Weert J, Smidt H, Boschker HTS, de Vos WM, Schraa G, Stams AJM (2003). Description of *Sulfurospirillum halorespirans* sp. nov., an anaerobic, tetrachloroethene-respiring bacterium, and transfer of *Dehalospirillum multivorans* to the genus *Sulfurospirillum* as *Sulfurospirillum multivorans* comb. nov. Int J Syst Evol Microbiol.

[b74] Macalady JL, Dattagupta S, Schaperdoth I, Jones DS, Druschel GK, Eastman D (2008). Niche differentiation among sulfur-oxidizing bacterial populations in cave waters. ISME J.

[b52] Macbeth TW, Cummings DE, Spring S, Petzke LM, Sorenson KS (2004). Molecular characterization of a dechlorinating community resulting from *in situ* biostimulation in a trichloroethene-contaminated deep, fractured basalt aquifer and comparison to a derivative laboratory culture. Appl Environ Microbiol.

[b11] Marcano N (2011).

[b53] Magot M, Ollivier B, Magot M (2005). Indigenous microbial communities in oil fields. Petroleum Microbiology.

[b54] Marmur J (1961). A procedure for the isolation of deoxyribonucleic acid from micro-organisms. J Mol Biol.

[b56] Miller WG, Parker CT, Rubenfield M, Mendz GL, Wösten MMSM, Ussery DW (2007). The complete genome sequence and analysis of the Epsilonproteobacterium *Arcobacter butzleri*. PLoS ONE.

[b57] Nazina TN, Shestakova NM, Grigoryan AA, Mikhailova EM, Tourova TP, Poltaraus AB (2006). Phylogenetic diversity and activity of anaerobic microorganisms of high-temperature horizons of the Dagang oil field (P.R. China). Microbiologiya.

[b58] Nelson DK, Ohene-Adjei S, Hu FS, Cann IKO, Mackie RI (2007). Bacterial diversity and distribution in the Holocene sediments of a northern temperate lake. Microb Ecol.

[b59] Nold SC, Zajack HA, Biddanda BA (2010). Eukaryal and archaeal diversity in a submerged sinkhole ecosystem influenced by sulfur-rich, hypoxic groundwater. J Great Lakes Res.

[b60] Oldenburg TBP, Larter S, Adams J, Clements M, Hubert C, Rowan A (2009). Methods for recovery of microorganisms and intact microbial polar lipids (IPLs) from oil–water mixtures: laboratory experiments and natural well-head fluids. Anal Chem.

[b61] Ollivier B, Cayol JL, Ollivier B, Magot M (2005). The fermentative, iron-reducing, and nitrate-reducing microorganisms. Petroleum Microbiology.

[b85] Ollivier B, Fardeau ML, Cayol JL, Magot M, Patel BKC, Prensier G, Garcia JL (1998). *Methanocalculus halotolerans* gen. nov., sp. nov., isolated from an oil-producing well. Int J Syst Evol Microbiol.

[b86] Orphan VJ, Taylor LT, Hafenbradl D, Delong EF (2000). Culture-dependent and culture-independent characterization of microbial assemblages associated with high-temperature petroleum reservoirs. Appl Environ Microbiol.

[b130] Pham VD, Hnatow LD, Zhang S, Fallon RD, Jackson SC, Tomb JF (2009). Characterizing microbial diversity in production water from an Alaskan mesothermic petroleum reservoir with two independent molecular methods. Environ Microbiol.

[b65] Porter ML, Engel AS (2008). Diversity of uncultured *Epsilonproteobacteria* from terrestrial sulfidic caves and springs. Appl Environ Microbiol.

[b66] Pruesse E, Quast C, Knittel K, Fuchs B, Ludwig W, Peplies J, Glöckner FO (2007). SILVA: a comprehensive online resource for quality checked and aligned ribosomal RNA sequence data compatible with ARB. Nucleic Acids Res.

[b13] Rowan AK, Snape JR, Fearnside D, Barer MR, Curtis TP, Head IM (2003). Composition and diversity of ammonia-oxidising bacterial communities in wastewater treatment reactors of different design treating identical wastewater. FEMS Microbiol Ecol.

[b91] Sakai S, Imachi H, Sekiguchi Y, Tseng IC, Ohashi A, Harada H, Kamagata Y (2009). Cultivation of methanogens under low-hydrogen conditions by using the coculture method. Appl Environ Microbiol.

[b67] Schink B, Kremer DR, Hansen TA (1987). Pathway of propionate formation from ethanol in *Pelobacter propionicus*. Arch Microbiol.

[b68] Schlegel ME, McIntosh JC, Bates BL, Kirk MF, Martini AM (2011). Comparison of fluid geochemistry and microbiology of multiple organic-rich reservoirs in the Illinois Basin, USA: evidence for controls on methanogenesis and microbial transport. Geochim Cosmichim Acta.

[b94] Scholz-Muramatsu H, Neumann A, Messmer M, Moore E, Diekert G (1995). Isolation and characterization of *Dehalospirillum multivorans* gen. nov., sp. nov., a tetrachloroethene-utilizing, strictly anaerobic bacterium. Arch Microbiol.

[b131] Schubotz F, Wakeham SG, Lipp JS, Fredericks HF, Hinrichs KU (2009). Detection of microbial biomass by intact polar membrane lipid analysis in the water column and surface sediments of the Black Sea. Environ Microbiol.

[b132] Sette LD, Simioni KCM, Vasconcellos SP, Dussan LJ, Neto EVS, Oliveira VM (2007). Analysis of the composition of bacterial communities in oil reservoirs from a southern offshore Brazilian basin. Antonie Van Leeuwenhoek.

[b97] Shimizu S, Akiyama M, Naganuma T, Fujioka M, Nako M, Ishijima Y (2007). Molecular characterization of microbial communities in deep coal seam groundwater of northern Japan. Geobiology.

[b98] Simankova MV, Kotsyurbenko OR, Lueders T, Nozhevnikova AN, Wagner B, Conrad R, Friedrich MW (2003). Isolation and characterization of new strains of methanogens from cold terrestrial habitats. Syst Appl Microbiol.

[b73] Strąpoć D, Picardal FW, Turich C, Schaperdoth I, Macalady JL, Lipp JS (2008). Methane-producing microbial community in a coal bed of the Illinois basin. Appl Environ Microbiol.

[b140] Strausz OP, Lown EM (2003). The Chemistry of Alberta Oil Sands Bitumens and Heavy Oils.

[b75] Sturt HF, Summons RE, Smith K, Elvert M, Hinrichs KU (2004). Intact polar membrane lipids in prokaryotes and sediments deciphered by high-performance liquid chromatography/electrospray ionization multistage mass spectrometry – new biomarkers for biogeochemistry and microbial ecology. Rapid Commun Mass Spectrom.

[b77] Tamura K, Dudley J, Nei M, Kumar S (2007). *MEGA4*: Molecular Evolutionary Genetics Analysis (MEGA) software version 4.0. Mol Biol Evol.

[b78] Telang AJ, Ebert S, Foght JM, Westlake DWS, Jenneman GE, Gevertz D, Voordouw G (1997). Effect of nitrate injection on the microbial community in an oil field as monitored by reverse sample genome probing. Appl Environ Microbiol.

[b79] Thamdrup B, Rosselló-Mora R, Amann R (2000). Microbial manganese and sulfate reduction in Black Sea shelf sediments. Appl Environ Microbiol.

[b80] Tseng H-Y, Person M, Onstott TC (1998). Hydrogeologic constraint on the origin of deep subsurface microorganisms within a Triassic basin. Water Resour Res.

[b81] US Patent 2010/0047793 A1

[b83] Vetriani C, Tran HV, Kerkhoff LJ (2003). Fingerprinting microbial assemblages from the oxic/anoxic chemocline of the Black Sea. Appl Environ Microbiol.

[b84] Voordouw G, Niviere V, Ferris GF, Fedorak PM, Westlake DWS (1990). Distribution of hydrogenase genes in *Desulfovibrio* spp. and their use in identification of species from the oil field environment. Appl Environ Microbiol.

[b133] Voordouw G, Armstrong SM, Reimer MF, Fouts B, Telang AJ, Shen Y, Gevertz D (1996). Characterization of 16S rRNA genes from oil field microbial communities indicates the presence of a variety of sulfate-reducing, fermentative, and sulfide oxidizing bacteria. Appl Environ Microbiol.

[b134] Wakeham SG, Amann R, Freeman KH, Hopmans EC, Jørgensen BB, Putnam IF (2007). Microbial ecology of the stratified water column of the Black Sea as revealed by a comprehensive biomarker study. Org Geochem.

[b87] Watanabe K, Watanabe K, Kodama Y, Syutsubo K, Harayama S (2000). Molecular characterization of bacterial populations in petroleum-contaminated groundwater discharged from underground crude oil storage cavities. Appl Environ Microbiol.

[b88] Watanabe K, Kodama Y, Kaku N (2002). Diversity and abundance of bacteria in an underground oil-storage cavity. BMC Microbiol.

[b89] Webster G, Rinna J, Roussel EG, Fry JC, Weightman AJ, Parkes RJ (2010). Prokaryotic functional diversity in different biogeochemical depth zones in tidal sediments of the Severn estuary, UK, revealed by stable-isotope probing. FEMS Microbiol Ecol.

[b90] Westerholm M, Roos S, Schnürer A (2010). *Syntrophaceticus schinkii* gen. nov., sp. nov., an anaerobic, syntrophic acetate-oxidizing bacterium isolated from a mesophilic anaerobic filter. FEMS Microbiol Lett.

[b135] Winderl C, Anneser B, Griebler C, Meckenstock RU, Lueders T (2008). Depth-resolved quantification of anaerobic toluene degraders and aquifer microbial community patterns in distinct redox zones of a tar oil contaminant plume. Appl Environ Microbiol.

[b116] Yashiro Y, Sakai S, Ehara M, Miyazaki M, Yamaguchi T, Imachi H (2011). *Methanoregula formicica* sp. nov., a methane-producing archaeon isolated from methanogenic sludge. Int J Syst Evol Microbiol.

[b93] Youssef N, Elshahed MS, McInerney MJ (2009). Microbial processes in oil fields: culprits, problems, and opportunities. Adv Appl Microbiol.

[b136] Zengler K, Richnow HH, Rosselló-Mora R, Michaelis W, Widdel F (1999). Methane formation from long-chain alkanes by anaerobic microorganisms. Nature.

[b119] Zhao Y, Boone DR, Mah RA, Boone JE, Xun L (1989). Isolation and characterization of *Methanocorpusculum labreanum* sp. nov. from the LaBrea tar pit. Int J Syst Bacteriol.

