# Supplementary material for: Massive dominance of Epsilonproteobacteria in formation waters from a Canadian oil sands reservoir containing severely biodegraded oil
Source: Environ Microbiol. 2012 Feb;14(2):387–404. doi: 10.1111/j.1462-2920.2011.02521.x (PMC3490369; doi:10.1111/j.1462-2920.2011.02521.x)
Supplement: Supplementary file 1 — Additional Supporting Information may be found in the online version of this article: Table S1. PCR primers used for microbial community analyses. Please note: Wiley-Blackwell are not responsible for the content or functionality of any supporting materials supplied by the authors. Any queries (other than missing material) should be directed to the corresponding author for the article. [file emi0014-0387-SD1.doc]

| **target**  **group** | **primer**  **names** | **forward primer (5’ to 3’)** | **reverse primer (5’ to 3’)** | **downstream**  **analysis** | **template**  **DNA used** | **Reference** |
| --- | --- | --- | --- | --- | --- | --- |
|  |  |  |  |  |  |  |
| *Bacteria* | 9f/1545r | GAGTTTGATCCTGGCTCAG | AGAAAGGAGGTGATCCAGCC | clone library | Calgary/  JCVI^1^ |  |
| *Bacteria* | 8f/1542r | AGAGTTTGATCCTGGCTCAG | AAGGAGGTGATCCAGCCGCA | clone library;  nested PCR and DGGE | Newcastle | Edwards et al., 1989 |
| *Bacteria* | inosine-341f/ 1492r | CCTACGGGIGGCIGCA | GGTTACCTTGTTACGACTT | clone library;  nested PCR and DGGE | Newcastle | Watanabe et al., 2002 |
| *Bacteria* | 341f-GC/534r | CGCCCGCCGCGCGCGGCGGGGCGGGCGGGGGCACGGGGGG-CCTACGGGAGGCAGCAG | ATTACCGCGGCTGCTGG | 2^nd^ round PCR and DGGE | Newcastle | Muyzer et al., 1993 |
| *Bacteria* | inosine-341f-GC/  534r | CGCCCGCCGCGCGCGGCGGGCGGGGCGGGGGCACGGGGGG-CCTACGGGIGGCIGCA | ATTACCGCGGCTGCTGG | 2^nd^ round PCR and DGGE | Newcastle | Watanabe et al., 2001 |
| *Archaea* | arch8f/arch1492r | TCCGGTTGATCCTGCC | GGCTACCTTGTTACGACTT | clone library | Calgary/  JCVI |  |
| *Archaea* | arch46/arch1017 | YTAAGCCATGCRAGT | GGCCATGCACCWCCTCTC | clone library;  nested PCR and DGGE | Newcastle | Øvreås et al., 1997;  Barns et al., 1994 |
| *Archaea* | arch344-GC/ Uni522r^x^ | CGCCCGCCGCGCGCGGCGGGCGGGGCGGGGGCACGGGGGGAGGGG-HGCAGCAGGCGCGA | GWATTACCGCGGCKGCTG | DGGE | Newcastle | Gray at al., 2002;  Amann et al., 1995 |

**Table S1**. PCR primers used for microbial community analyses.

^1^ The J. Craig Venter Institute (JCVI) constructed clone libraries by amplifying DNA that was extracted at the University of Calgary.

**References for Supporting Information**

Amann, R., Ludwig, W., and Schleifer K-H. (1995) Phylogenetic identification and in situ detection of individual microbial cells without cultivation. *Microbiol. Rev*. 59: 143-169.

Barns, S., Fundyga, R.E., Jeffries, M.W., and Pace, N.R. (1994) Remarkable archaeal diversity detected in a Yellowstone National Park hot spring environment. *Proc. Nat. Acad. Sci*. 91: 1609-1613.

Gray, N.D., Miskin, I.P., Kornilova, O., Curtis, T.P., Head, I.M. (2002) Occurrence and activity of *Archaea* in aerated activated sludge wastewater treatment plants. *Environ. Microbiol*. 4: 158-168.

Edwards, U., Rogall, T., Blöcker, H., Emde, M., Böttger, E.C. (1989) Isolation and direct complete nucleotide determination of entire genes. Characterisation of a gene coding for 16S ribosomal RNA. *Nucl. Acids Res*. 17:7843-7853.

Muyzer, G., De Waal, E.C., and Utterlinden, A.G. (1993) Profiling of complex microbial populations by denaturing Gradient Gel Electrophoresis analysis of Polymerase Chain Reaction-amplified genes coding for 16S rRNA. *Appl. Environ. Microbiol*. 59: 695-700.

Øvreås, L., Forney, L., Daae, F.L., and Torsvik, V. (1997) Distribution of bacterioplankton in meromictic lake Sælenvannet, as determined by denaturing gradient gel electrophoresis of PCR-amplified gene fragments coding for 16S rRNA. *Appl. Environ. Microbiol*. 63: 3367-3373.

Watanabe, K., Kodama, Y., Harayama, S. (2001) Design and evaluation of PCR primers to amplify bacterial 16S ribosomal DNA fragments used for community fingerprinting. *J. Microbiol. Meth*. 44: 253-262.

Watanabe, K., Kodama, Y., Kaku, N. (2002) Diversity and abundance of bacteria in an underground oil-storage cavity. *BMC Microbiol*. 2:23.
